# Public Health Work

**Published:** 1907-09-07

**Authors:** 


### PUBLIC HEALTH WORK.

The Diploma in Public Health is a qualification which will prove of undoubted service to the graduate who desires to apply for an appointment under the Health authorities.

A medical officer of health of any county, or of any single or combined district with a population at the last census of 50,000 or more, or of any metropolitan district, or the deputy of any such officer, must be legally qualified for the practice of medicine, surgery, and midwifery, and must either be registered in the "Medical Register" as a holder of a diploma in sanitary science, public health, or State medicine, under Section 21 of the Medical Act of 1886, or must have been, during three consecutive years preceding the year 1892, a medical officer of a district or districts in London or elsewhere with a population according to the last census of not less than 20,000, or must have been, before the passing of the Local Government Act of 1888, for not less than three years, a medical officer or inspector of the Local Government Board.

It is advisable to obtain the diploma early, and then to try and obtain an assistantship to an officer in charge of a large borough district. Diplomas are granted by nearly all examining bodies. The Conjoint Board of England grants a diploma in public health. The Universities of Oxford, Cambridge, Durham, Liverpool, Manchester, Sheffield, and Leeds grant diplomas in hygiene and public health to their own graduates or to graduates of sister universities, and in some instances also grant diplomas to qualified practitioners who may not be graduates. The requirements for each diploma varies, but particulars may usually be obtained on application to the various registrars. The most popular diplomas appear to be the Cambridge D.P.H. and those granted by the University of Durham. For both the candidate must be a registered medical practitioner and must have attended special courses of instruction at recognised centres or with recognised teachers for a specified period.

The Conjoint Board of Scotland requires candidates to produce evidence of attendance (subsequent to obtaining a registrable qualification) for six months at a Public Health Laboratory, and for six months under a Medical Officer of Health of a county or of a large urban district. There are two examinations, and candidates may present themselves for both of them at one period or for either examination separately. The fee is £12 12s., or £6 6s. in respect of each examination; and candidates referred are re-admitted on a fee of £3 3s. in respect of each examination. The examination is held in Edinburgh or in Glasgow, there being two periods of examinations yearly—October and May. Applications for examination in Edinburgh to be sent to Mr. James Robertson, 54 George Square, Edinburgh; and in Glasgow to Mr. Alexander Duncan, B.A., LL.D., 242, St. Vincent Street, Glasgow, not later than fourteen days before the examination day. The University of Dublin confers degrees after examinations upon M.D.'s or graduates in Medicine and Surgery of Dublin, Oxford, or Cambridge, and the Royal University only on graduates of Medicine of that University. The Conjoint Examining Board grants diplomas after examination to candidates who have complied with the regulations of the General Medical Council.

The University of Edinburgh grants a diploma in tropical medicine and hygiene. The examinations are held in January and July. The fees for the first and any subsequent appearance are: Practical bacteriology, £1 1s.; diseases of tropical climates, £1 1s.; tropical hygiene, £1 1s.; tropical clinical medicine, £1 1s.; total, £4 4s.

